# Does a Suprascapular Nerve block reduce chronic shoulder pain at 3 months compared to standard non-operative care? A systematic review

**DOI:** 10.1007/s00590-025-04287-4

**Published:** 2025-04-21

**Authors:** Sean David Scattergood, Abdul Hassan, Mark Williams

**Affiliations:** 1 University of Bristol, Bristol, UK; 2 North Bristol NHS Trust, Bristol, UK

**Keywords:** Suprascapular nerve block, Shoulder, Chronic pain, Non-operative care

## Abstract

**Purpose:**

Chronic shoulder pain affects 2.4–30% of adults at any given time because of a wide variety of underlying pathologies. Treatment of chronic shoulder pain should attempt to address the underlying cause, if possible, either through surgical or non-surgical means. Routine non-operative care involves analgesia, physiotherapy and perhaps injection of corticosteroid at the appropriate site. A suprascapular nerve block (SSNB) is a minimally invasive, low-risk procedure which offers analgesia for patients. This review evaluates the effectiveness of SSNB in reducing shoulder pain at a 3-month follow-up, in comparison to standard non-operative care.

**Methods:**

A literature search was conducted across Medline, Embase, Cochrane, ISRCTN and clinicaltrial.gov databases from inception to November 2024. Ninety publications were screened by abstract followed by full text, by the authors against inclusion criteria, and risk of bias was assessed using the RoB 2 tool.

**Results:**

Five randomised studies were included for analysis, presenting a heterogeneous mix of intervention, study populations and outcome measures. The two studies performing SSNB with local anaesthetic (LA) and corticosteroid found a significant reduction in pain at 3 months. In contrast, compared to studies using LA alone did not demonstrate the same level of efficacy.

**Conclusion:**

Suprascapular nerve block is an effective analgesic option for chronic shoulder pain, which can be offered to patients as part of a shared decision-making approach. While studies suggest efficacy of combined LA and corticosteroid, limitations such as heterogeneity, variability in result reporting and short follow-up periods reduce the strength of the evidence.

## Introduction

Chronic shoulder pain is a common presentation to tertiary shoulder clinics, with a prevalence in adults of 2.4–30% [1, 2]. In England alone, approximately 1.5 million general practitioner (GP) consultations a year are related to shoulder pain, with an estimated societal cost to the United Kingdom (UK) of £100 million [3]. Multiple pathologies, sometimes simultaneously, may lead to the development of a chronic pain state defined in this study as pain persisting longer than 3 months [4]. They may be intra-articular in nature such as inflammatory arthritis, osteoarthritis or adhesive capsulitis. Alternatively, causes may be related to the sub-acromial space such as sub-acromial pain syndrome or rotator cuff injury pathologies. Treatment of chronic shoulder pain should attempt to address the underlying cause, if possible, either through surgical or non-surgical means. Many patients suffering pain which could be treated with surgical intervention may be unsuitable to undergo surgery due to a various reasons including multimorbidity, lack of a clear surgical target, unwillingness to assume surgical risks or an inability to manage postoperative rehabilitation. Standard care for these patients involves oral analgesia, physiotherapy and targeted corticosteroid injections with variable efficacy [5, 6]. Chronic shoulder pain remains a major cause of disability, particularly in the elderly [3, 7].

A suprascapular nerve block (SSNB) offers an alternative pain management approach by targeting the suprascapular nerve, which supplies 70% of the sensory innervation to the shoulder and acromioclavicular joints [8–10]. A suprascapular nerve block is a safe, efficient, low-risk procedure which may provide long-lasting analgesia [2, 11]. It may be performed in an outpatient procedure room with sterile equipment, with or without ultrasound (US) guidance [12]. This study reviews the current evidence on the efficacy of SSNB in relieving chronic shoulder pain, compared to standard non-operative care. The null hypothesis is that SSNB provides no greater pain relief than standard care after a minimum of 3 months.

## Methods

### Search methods & strategy

**Fig. 1:**
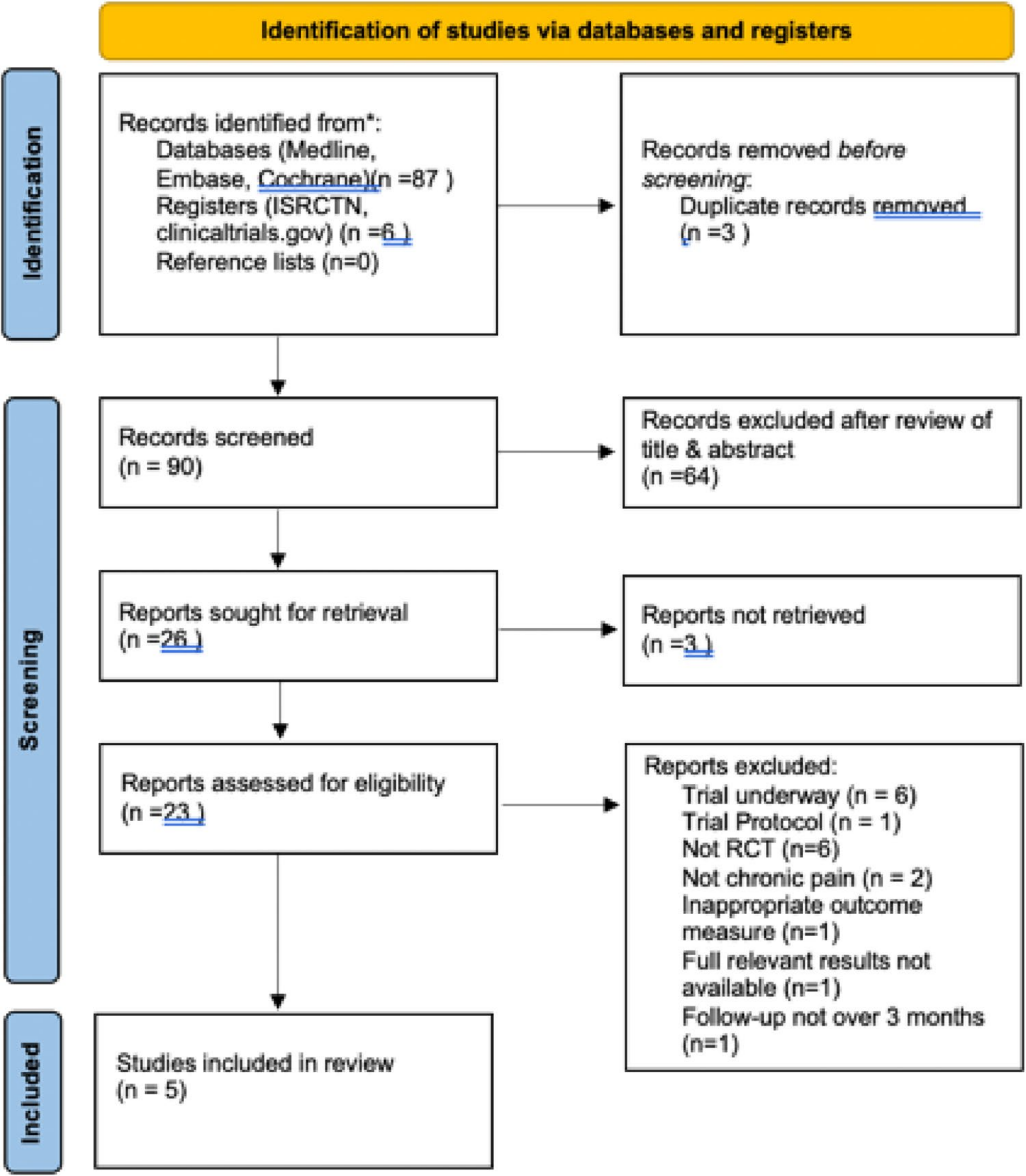
PRISMA flow diagram

A literature search was performed of Medline, Embase, Cochrane, International Standard Randomised Controlled Trial Number (ISRCTN) and clinicaltrial.gov databases from inception to November 2024. Search strategy consisted of a selection of MeSH headings & free search terms and is available in Appendix 1. Reference lists of analysed articles were searched for additional studies. A PRISMA flow diagram can be seen in Fig. 1.

### Review and quality assessment

**Fig. 2:**
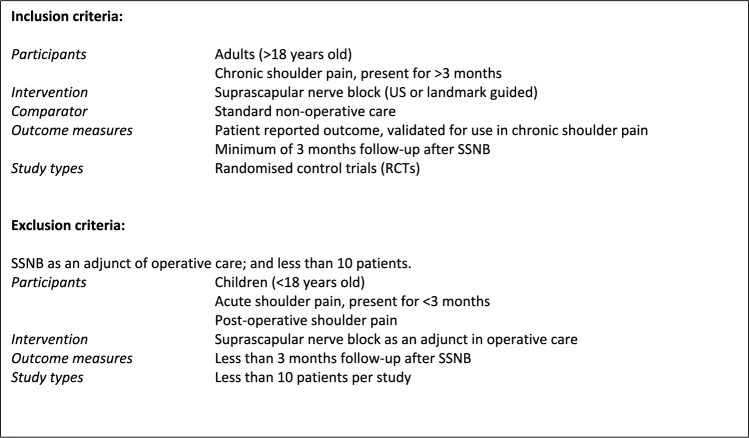
Inclusion and exclusion criteria

Screening of articles by abstract was undertaken by the first author (S.S). Full-text articles were then assessed for eligibility using the predefined inclusion and exclusion criteria (Fig. 2) by two authors independently (S.S and A.H). Where there were discrepancies in decisions made between the two reviewers, these articles were reviewed by the senior author (M.W) who made a final decision. Full-text articles that met the inclusion criteria were included in this review.

A risk of bias assessment was undertaken using the Cochrane ROB2 tool by (S.S) and (A.H), with any discrepancies escalated to the third author for a decision (M.W) [13].

### Data extraction and synthesis

Data on pathology or diagnosis, age of participants, SSNB technique, SSNB injection components and the routine care comparator group were extracted into the predefined extraction tables.

## Results

### Search results

Our search returned ninety-three studies, from which we removed 3 duplicates. Ninety studies were screened by title and abstract, 26 studies were sought for retrieval, but 3 were not retrieved. Twenty-three studies were assessed by full text, with five randomised studies included for analysis. Articles excluded were recorded at each stage of the screening process and reported in the Preferred Reporting Items for Systematic Reviews (PRISMA) flow diagram (Fig. 1).

### Study characteristics

**Fig. 3:**
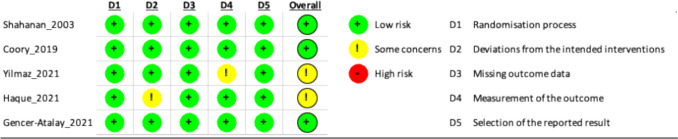
Risk of bias assessment using the ROB 2 tool

We included 5 studies from across the world including Australia and Turkey, which are summarised in Table 1 [10, 14–17]. Table 1 shows that the studies differ significantly across several key aspects: shoulder pathology, SSNB technique (ultrasound-guided vs. anatomical landmark), composition of the injected solution for SSNB, comparator groups and outcome measures. Included studies range in size from 40 patients to 108, all with at least 3-month follow-up. All studies included only adult patients (Fig. 3).

**Table 1:** Summary of studies [10, 14–17]

Study	Pathology	Technique	SSNB Components	Routine Care	Number			Outcome	Follow-up (months)
					Overall	SSNB	Routine Care		
Coory [14]	Rotator cuff tear seen on MRI or USS	USS	9 mL 1% ropivacaine & 1 mL of betamethasone	Sub-acromial injection (components same as SSNB)	43	21	22	VAS	3
Gencer-Atalay [15]	Adhesive capsulitis	USS	5 mL 0.5% bupivacaine	Intra-articular GHJ injection (1 mL of betamethasone and 4 mL of 0.5% bupivacaine)	40	20	20	SPADI	12
Haque [16]	Adhesive capsulitis	Landmark	10 mL 0.5% bupivacaine	Intra-articular GHJ injection (40 mg triamcinolone and 1 mL 2% lidocaine)	86	43	43	SPADI	3
Shanahan [10]	Osteoarthritis or rheumatoid arthritis	Landmark	10 mL 0.5% bupivacaine & 40 mg methylprednisolone	Placebo injection (5 mL 0.9% saline subcutaneously)	108	52	52	SPADI	3
Yilmaz [17]	SIS, other diagnoses excluded via MRI	Landmark	4 mL 0.5% bupivacaine & 1 mL 0.9% saline	Sub-acromial injection (4 mL 2% lidocaine & 1 mL triamcinolone)	66	33	33	VAS	3

*MRI*; Magnetic resonance imaging, *USS*; Ultrasound scan, *AC*; Adhesive capsulitis, *OA*; Osteoarthritis, *RA*; Rheumatoid arthritis, *VAS*; Visual analogue score, *SPADI*; Shoulder pain and disability index, *SSNB*; Suprascapular nerve block, *SIS*; Sub-acromial impingement syndrome, *GHJ*; Glenohumeral joint

### Study populations and cause of chronic pain

A variety of diagnoses which may cause chronic shoulder pain were included in this review. Participants in the study from Coory et al. included 43 adults > 45 years with a symptomatic rotator cuff tear confirmed on MRI or USS [14]. Gencer-Atalay and Haque et al. investigated 40 and 86 adults respectively with adhesive capsulitis, but Gencer-Atalay only looked at participants between the age of 30 and 70 years [15, 16]. Shanahan et al. study investigated 108 participants with either rheumatoid or osteoarthritis, and Yilmaz et al. looked at 66 adults with sub-acromial impingement syndrome with other causes of pain excluded on MRI [10, 17].

### SSNB technique & medication components

In studies from Coory and Gencer-Atalay et al., SSNB was performed under ultrasound guidance, with SSNB being performed in the other three studies using an anatomical landmark technique [10, 14–16]. Three studies used 0.5% bupivacaine alone to perform SSNB, with Haque et al. injecting 10 mL, Gencer-Atalay et al. 5 mL and Yilmaz 4 mL, respectively [15–17]. Coory et al. performed SSNB using 9 mL of 1% ropivacaine as local anaesthetic combined with 1 mL of betamethasone [17]. Shanahan et al. administered SSNB with 10 mL of 0.5% bupivacaine & 40 mg methylprednisolone, after infiltrating subcutaneously with 2 mL 1% lidocaine [10].

### Routine care (comparator group)

In studies from Coory et al. and Yilmaz et al., routine care consisted of a sub-acromial injection, a combination of 9 mL 1% ropivacaine & 1 mL of betamethasone for Coory and 4 mL 2% lidocaine & 1 mL triamcinolone for Yilmaz et al. [14, 17]. Gencer-Atalay and Haque et al. performed an glenohumeral intra-articular injection, Gencer-Atalay using 1 mL of betamethasone and 4 mL of 0.5% bupivacaine and Haque et al. using 40 mg triamcinolone and 1 mL 2% lidocaine [15, 16]. Shanahan et al. used a placebo injection of 5 mL 0.9% saline injected subcutaneously after infiltration of 2 mL 1% lidocaine as in their treatment group [10].

### Outcome measures

Gencer-Atalay et al., Haque et al. and Shanahan et al. all used the shoulder pain and disability index (SPADI) scoring system as primary outcome, Yilmaz et al. used the visual analogue score (VAS) as primary outcome and Coory et al. assessed the VAS as a secondary outcome [10, 14–17]. Four studies assessed outcomes at 3 months after SSNB, but Gencer-Atalay extended follow-up to 12 months post intervention [10, 14–17]. Both the VAS and SPADI are validated for use in chronic shoulder conditions [18–21]. The minimum clinically important difference (MCID) for the SPADI is 10, and for VAS estimates range between 1.4 and 3, but we have chosen to use an MCID of 2.2 based on most recent evidence [18, 20–22].

Significant heterogeneity exists across the five studies in several key aspects: the specific shoulder pathology treated with SSNB, the technique employed (ultrasound-guided vs. anatomical landmark), and the composition of the injected solutions for both treatment and comparator groups varies considerably between studies. Three studies used local anaesthetic alone to perform SSNB, and two studies used a mixture of local anaesthetic and corticosteroid.

Due to the heterogeneity present across outcome measures, follow-up length, selected interventions and comparator groups, meta-analysis was deemed inappropriate. Therefore, a systematic review was conducted.

### Summary of study results

A summary of results for studies using SPADI as the primary outcome is seen in Table 2, and for studies reporting VAS in Table 3.

**Table 2:** Summary of results reporting SPADI score

Study	SSNB			*P*-value	Routine Care			*P*-value
	Before	3 months	12 months		Before	3 months	12 months	
Gencer-Atalay [15]	80.8 (70.7–85.5)	19.2 (14.9–24.0)	8.5 (5.9–21.7)		83.1 (72.5–86.2)	16.2 (10.6–33.1)	4.6 (4.8 -19.6)	
Haque [16]	52.6	9.6		0.000	51.4	11.7		0.000
Shanahan [10]	68.1 (63.7–72.6)	55.5 (49.3–61.7)			66.5 (61.4–71.6)	63.9 (57.8– 69.8)		

*SSNB*; Suprascapular nerve block, *SPADI*; Shoulder pain and disability index, *CI*; Confidence interval

*Gencer-Atalay *et al*. And Shanahan *et al. *presented as mean (95% CI)*

*Haque *et al*. presented as mean (standard deviation), if standard deviation available*

*P-values reported where available*

**Table 3:** Summary of results reporting visual analogue score (VAS)

Study	Follow-up (months)	Before		*P*-value	After		*P*-value	95% CI
		SSNB	Routine		SSNB	Routine		
Coory [14]	3	7.8 (4.2)	7.4 (4.0)	0.753	9.9 (3.3)	7.3 (4.3)	0.315	n/a
Yilmaz [17]	3	8.5 (0.9)	8.6 (0.99)	0.334	1.5 (0.6)	3.1 (1.1)	< 0.001	n/a

*SSNB*; Suprascapular nerve block

*Data presented as mean (standard deviation)*

*P-values reported where available*

*95% confidence intervals reported where available*

Haque et al. found a difference in total SPADI score of 43 points (*P* = 0.002) at 3-month follow-up for SSNB, compared to 39.7 points for routine care, giving a difference in mean improvement of 3.3 points (*p* = 0.002) [16].

Gencer-Atalay et al. found a difference in mean total SPADI score from baseline to 3 months of 61.6 and at 12 months of 72.3 for the intervention group (SSNB) [15]. Gencer-Atalay report a difference in mean total SPADI score from baseline to 3 months of 66.9 and at 12 months of 78.5 for routine care, showing a slightly better improvement in SPADI score for routine care at both follow-up timepoints [15].

Shanahan et al. shows an improvement of 12.6 (SD 19.3) point's total SPADI score at 3 months for SSNB group, compared to an improvement of 2.6 for the placebo group. This gives a difference in mean improvement between SSNB and placebo groups of 10 points (95%CI 3.6 to 18.2) [10].

Coory et al. and Yilmaz et al. both use VAS as an outcome measure [14, 17]. Coory et al. report a mean VAS for each participant group, before and after intervention; with an improvement of 2.1 points for participants after a SSNB [14]. Yilmaz et al. also report mean and standard deviation of VAS for intervention and comparator groups, before and after injection; they found a VAS mean improvement of 7 at 3 months for SSNB compared to 5.5 for standard care [17].

#### Critical appraisal

A risk of bias assessment using the Cochrane risk of bias-2 framework (ROB 2) identified some methodological flaws with Yilmaz et al. and Haque et al. mainly pertaining to blinding of the patients or outcome assessors [13, 16, 17]. Included studies are of an acceptable standard, but Coory et al., Gencer-Atalay et al. & Shanahan et al. used a sealed envelope randomisation process which could have led to allocation bias [10, 14, 15]. Yilmaz et al. presented results that were difficult to interpret due to unclear writing in the result section, raising concerns of reporting bias [17].

## Discussion

The suprascapular nerve has three sensory branches: medial & lateral sub-acromial and the posterior glenohumeral, which collectively innervate the sub-acromial bursa, glenohumeral joint capsule and acromioclavicular joint [12]. It is thought that blockade of these sensory signals would offer pain relief to the patient, irrespective of the specific pathology causing pain, assuming that the above anatomical structures are involved [2].

Table 1 shows many differences across included studies, from outcome measures to shoulder pathology being treated with SSNB, to SSNB technique and constituents. Two studies investigated patients with adhesive capsulitis (AC), one study rotator cuff tears, one shoulder impingement (SIS) and one Osteoarthritis (OA) or rheumatoid arthritis (RA). Four studies compare SSNB to a steroid injection and one compared to placebo, three studies used anatomical landmarks to perform the SSNB and two performed using US guidance. The variety of SSNB technique and constituents in the studies included in this review is representative of the literature as whole: There is currently no accepted standard of SSNB technique and components [2]. The presence of this heterogeneity precludes meta-analysis. However, the variety of shoulder pathologies in the included studies reflects clinical practice in the UK, and the multiple different shoulder pathologies for which SSNB is offered in clinical practice.

We found that all three studies using SPADI score as their primary outcome showed a suggestion of superiority for SSNB over routine care, but only Shanahan et al. report a difference which meets the MCID for SPADI of 10 points [10, 15, 16]. It is important to interpret this finding with the knowledge that Shanahan et al. used a placebo injection as their comparator group, so it is possible that less of a difference between groups would be present if SSNB was compared to routine care such as an intra-articular steroid injection or physiotherapy [10]. Regarding studies reporting VAS for pain, Coory et al. reported an adjusted difference of 2.3 between treatment groups, and Yilmaz et al. of 1.5, both of which are at the level of, or just below the MCID we have chosen to use in this review [14].

Current literature suggests that SSNB is an effective method of pain relief for non-specific pain shoulder pain and is superior to physiotherapy and placebo, but similar to targeted injections of steroid [2, 4, 12, 23, 24]. Chang et al. performed a meta-analysis of 11 randomised control trials to investigate the effectiveness of SSNB with routine care in chronic shoulder pain [4]. They report that SSNB provide better pain relief compared to physiotherapy or placebo, and similar results to intra-articular injection of the GH joint at one-month follow-up [4]. In 2021, Jump et al. published a meta-analysis which included 6 studies investigating the impact of SSNB on pain in patients with frozen shoulder [12]. They report that pain levels were significantly reduced after SSNB compared with baseline, with no comparator group available and significant heterogeneity which may affect their analysis [12].

Two included studies (Coory et al. and Shanahan et al.) combine corticosteroid and local anaesthetic (LA) for SSNB, which is the current standard at our institution, with the other three studies infiltrating LA alone [10, 14–17]. It is believed that LA acutely interrupts pain transmission, and corticosteroid may have a more prolonged effect by reducing the release of inflammatory neuropeptides and cytokines to relieve entrapment and fibrosis [12]. Coory et al. compared SSNB to a sub-acromial steroid injection in patients with rotator cuff tear, and Shanahan et al. compared SSNB to a placebo injection with 0.9% saline in patients suffering OA or RA [10, 14]. These two studies found a significant efficacy for SSNB compared to the remaining other studies which did not. We postulate that this difference may be due to the inclusion of steroid.

Coory et al. findings of a mean improvement of 2.1 points on VAS at 3 months is in favour of superior analgesia with SSNB compared to a sub-acromial injection for chronic rotator cuff tear, but their reported p-values do not meet statistical significance [14]. Whilst this study was at generally classed as low risk of bias, it did suffer a potential bias during randomisation. Coory et al. fail to present a difference in means pre-procedure and post-procedure, or provide a p-value and 95% confidence intervals for a difference. By only reporting the primary outcome at follow-up, and not the mean improvement, the results are not adjusted for the pre-procedure score, which may lead to a misleading conclusion.

In Shanahan et al. study sample, 52% of participants (56/108) had degenerative disease, (degenerative disease refers to degenerative changes in the glenohumeral joint, acromioclavicular joint and/or rotator cuff), and 48% of patients had rheumatoid arthritis, which due to its autoimmune nature may not respond to a nerve block as effectively as non-inflammatory conditions [10]. Shanahan et al. compared SSNB to a placebo injection, which does not reflect standard care; it could be argued that this lead to less external validity and generalisability to UK practice for their study [10].

Two studies performed SSNB under US guidance, while three used an anatomical landmark technique. To date, there is no evidence that US-guided SSNB has superior outcomes compared to landmark-based techniques, but US use does decrease the risk of acute complications and local anaesthesia toxicity [12, 25, 26]. Given the widespread availability of US, it is increasingly difficult to justify not using an US-guided approach, as it may improve accuracy and minimise patient risk.

Overall, our findings are that SSNB is an effective method of treatment for chronic shoulder pain, with a suggestion that it may be superior to routine non-operative care: placebo, physiotherapy or targeted steroid injections (sub-acromial or GHJ intra-articular). This is in keeping with previous reviews and current literature [2, 4, 12, 23, 24]. Implications for clinicians are that SSNB is a sound treatment option for chronic shoulder pain with a variety of underlying pathologies, which is comparable or possibly superior to a combination of physiotherapy and steroid injection. Clinicians and patients should consider including SSNB in their management discussions as part of a shared decision-making process.

In conclusion, SSNB is an effective treatment option which offers analgesia to patients with chronic shoulder pain. A SSNB can be offered for a wide variety of shoulder pathology and can be an important treatment option, particularly in patients for whom a surgical intervention is within standard practice, but may not be possible due to other patient factors. Further research would be welcomed to help standardise SSNB technique, and quantify its analgesic efficacy & longevity compared to routine non-operative care, with follow-up longer than 3 months.

## Data Availability

No datasets were generated or analysed during the current study.

## Appendix 1

### Search Strategy

Ovid MEDLINE(R) < 1946 to present > 

1. Nerve Block/21259
2. Suprascapular.mp.1667
3. Shoulder Pain/ or Pain/ or Chronic Pain/175791
4. 1 and 2 and 3 92
5. Limit 4 to English language 87
